# Supplementary material for: The phased pan-genome of tetraploid European potato
Source: Nature. 2025 Apr 16;642(8067):389–97. doi: 10.1038/s41586-025-08843-0 (PMC12158759; doi:10.1038/s41586-025-08843-0)
Supplement: Supplementary file 5 — Supplementary Figs. 3–14, 20, 21, 23–27 and 34–44. [file 41586_2025_8843_MOESM5_ESM.zip › suppl_figure_3_to_14/suppl_figure_13_chr11/suppl_figure_13bm_44_I_Lum.pdf]

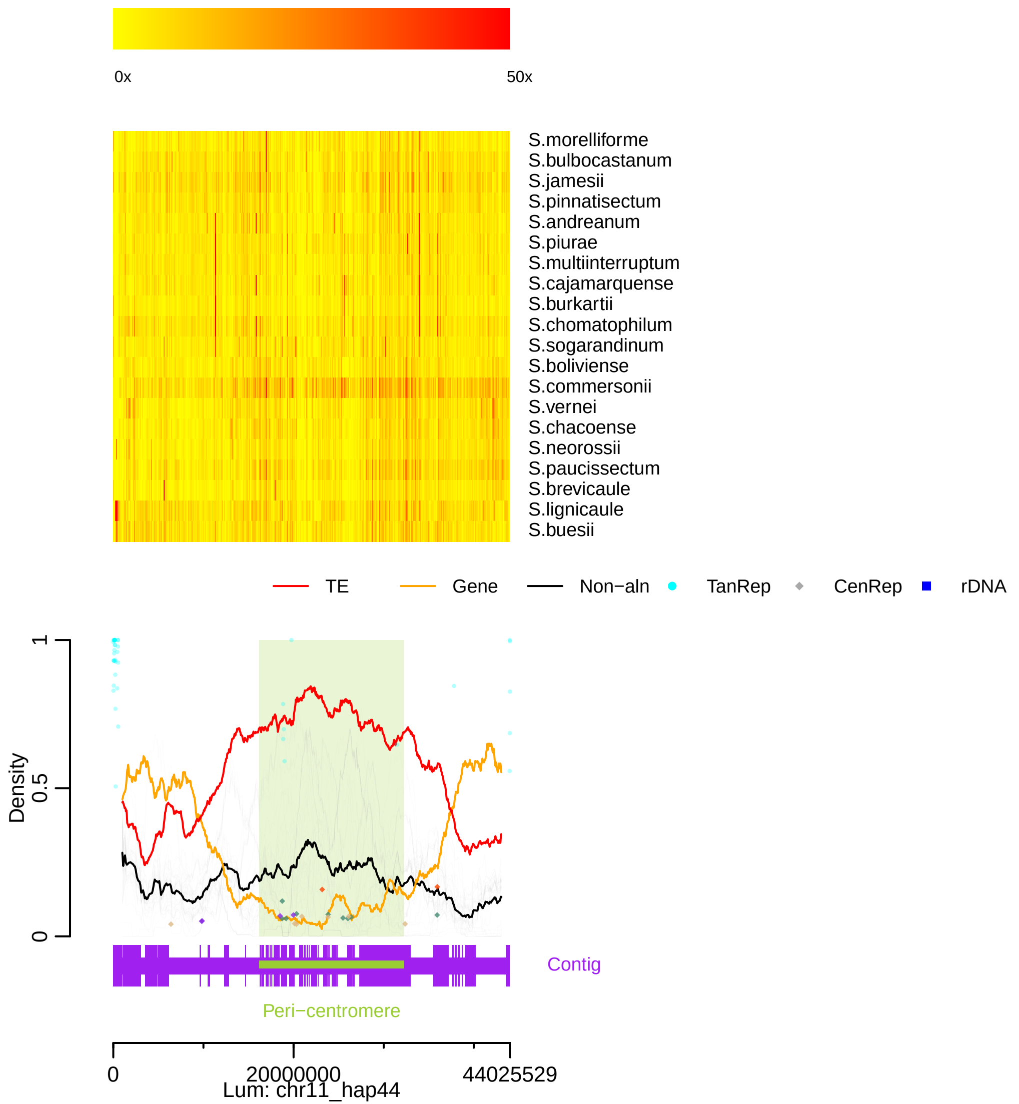

0x
50x
S.morelliforme
S.bulbocastanum
S.jamesii
S.pinnatisectum
S.andreanum
S.piurae
S.multiinterruptum
S.cajamarquense
S.burkartii
S.chomatophilum
S.sogarandinum
S.boliviense
S.commersonii
S.vernei
S.chacoense
S.neorossii
S.paucissectum
S.brevicaule
S.lignicaule
S.buesii
TE
Gene
Non−aln
TanRep
CenRep
rDNA
Density
1
0.5
0
Contig
Peri−centromere
0
20000000
44025529
Lum: chr11_hap44
